# Mapping the mechanical properties of paintings via nanoindentation: a new approach for cultural heritage studies

**DOI:** 10.1038/s41598-020-64892-7

**Published:** 2020-05-13

**Authors:** Mathilde Tiennot, Erik Paardekam, Davide Iannuzzi, Erma Hermens

**Affiliations:** 1 Conservation and Science Department, Rijksmuseum, Ateliergebouw, Hobbemastraat 22, 1071 ZC Amsterdam, Netherlands; 2 Department of Physics and Astronomy and LaserLab Amsterdam, Vrije Universiteit Amsterdam, De Boelelaan 1085, 1081 HV Amsterdam, Netherlands; 3 History of Art Department, University of Amsterdam, 1012 WX Amsterdam, Netherlands

**Keywords:** Mechanical properties, Characterization and analytical techniques

## Abstract

A comprehensive understanding of the behaviour of the heterogenous layers within the paint stratigraphies in historical paintings is crucial to evaluate their long term stability. We aim to refine nanoindentation as a new tool to investigate the mechanical behaviour of historical oil paints, by adapting the probes and the protocol already used in biomechanical research on soft tissues. The depth-controlled indentation profile performed with a spherical probe provides an evaluation of the non-linear viscoelastic behaviour of the individual layers in paint at local scale. The technique is non-destructive and guarantees the integrity of the surface after indentation. The mapping of elasticity demonstrates the properties’ heterogeneity of the composite material within the paint layers, as well as between the individual layers and their interfaces.

## Introduction

Natural aging, external mechanical impacts, and variations in relative humidity and temperature, as well as inherent chemical processes between pigments and binding media, can induce internal stresses in paint stratigraphies, which can lead to cracking and delamination^1–5^. A further understanding of the stress distribution within the different layers (support-ground-paint-varnish) is essential to evaluate crack initiation and propagation and to develop preventive conservation as well as paint consolidation methods^5^.

Classical mechanical investigation methods, such as tensile, bending or biaxial measurements, usually require large samples, and provide limited information on the viscosity or rheological behaviour of the paint film^1^. Furthermore, they can only be used to extract an overview on the macroscopic scale behaviour, giving no insight on how the heterogeneous properties of the various layers at the local scale may contribute to the overall mechanics. Methodologies investigating the local mechanical properties on small samples like cross-sections from paintings are, therefore, of major interest.

One methodology applied to evaluate the mechanical properties of paintings is nanoindentation, as proposed by Salvant *et al*.^4^ and Freeman *et al*.^6^. Salvant *et al*.^4^ used this method to investigate the hardness and reduced modulus of reconstructions as well as cross-sections of paintings^6^. presented results from nanoindentation used on reconstruction samples embedded in polyester resin. In these two studies, however, the samples were indented by means of a diamond Berkovitch tip - a method that is known to have severe limitations^7–9^ and, importantly, to induce mechanical plastic deformations that destroy the sample^4^.

Over the last couple of years, some of us have introduced a new indentation instrument that, in the field of life sciences, is nowadays recognized as a bridging tool between classical mechanical investigation methods and nanoindentation^10–22^. This new nanoindenter relies on a millimeter size cantilever, equipped with a microsphere glued at its free hanging end. The sphere is brought to contact with the sample, while the bending of the cantilever spring is monitored with an optical fiber based interferometer. Observing the deflection of the cantilever spring when the sphere is in contact with the sample, one can then assess the mechanical properties of the sample. The instrument allows one to gather maps of the local viscoelastic properties of materials with (sub)micron resolution without causing any damage to the indented point.

The optomechanical device proposed to carry out these specific measurements also guarantees more accurate measurements. The depth-controlled indentation profile allows more precise analysis and accurate dynamic mechanical analysis (DMA) at constant indentation depth, providing better estimation of both elastic and viscous moduli of the material^10–12,15^.

The aim of this paper is to demonstrate that this very same instrument can be used to successfully map the mechanical properties of painting samples. To achieve this goal, we present here two sets of measurements. The first set was performed on laboratory reconstructions, where we could test whether the instrument can perform well-controlled, reproducible analyses to assess the (non-linear) viscoelastic properties of the samples. The second set was carried out on a cross-section of a sample from a 17^*th*^-century painting, and was meant to illustrate the mapping capability of the instrument. From the results reported, one can conclude that our nanoindentation instrument truly represents a unique tool for cultural heritage studies.

## Materials

### Preparation of the laboratory reconstructions

Oil based paints are much stiffer than biological tissues^23^. It is thus expected that an instrument that has been designed to work on biological tissues may need significant adjustments before it can be used on paintings. To address this issue, we have conducted a series of experiments on mock-ups that mimic a stratigraphy typically found in Netherlandish 17^*th*^-century paintings, limiting it to the ground layers build-up. It should be noted that this is mainly intended as an historically informed mock-up of just the ground layers, which however is sufficient for our test.

**Figure 1:**
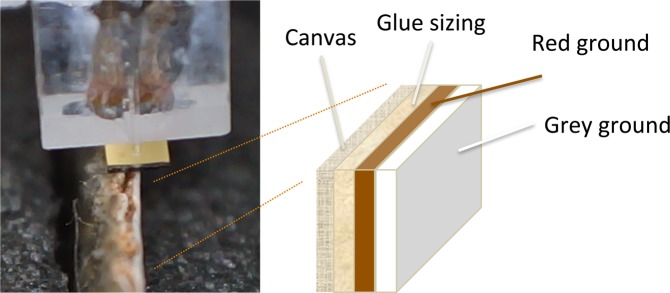
Laboratory reconstruction during the indentation measurement on the side and schematic representation of the three layers applied on the canvas.

To set up the samples, we prepared a stretched canvas with a size layer of rabbit skin glue in water. This was followed by a layer consisting of chalk (calcium carbonate) and a red earth pigment (iron oxide), mixed with raw linseed oil, resulting in a reddish brown ground layer. Then, a second grey ground layer made up of lead white, chalk and small amounts of yellow ochre, umber and ivory black in raw linseed oil, was applied. This ground layer system mimics an often found build-up of a double ground comprising a red layer followed by a grey layer (Fig. 1). Each layer was UV-dried during 24h after its application. For this research, two pieces were cut from the prepared canvas and were stored under controlled atmosphere at RH = 50% and T = 20 °C. Depth-controlled indentation profiles were adapted to the analysis of both the surface layer and on the cross-section of the sample, to measure their mechanical properties by following the protocol described in the Methods section.

### Rijksmuseum painting sample

**Figure 2:**
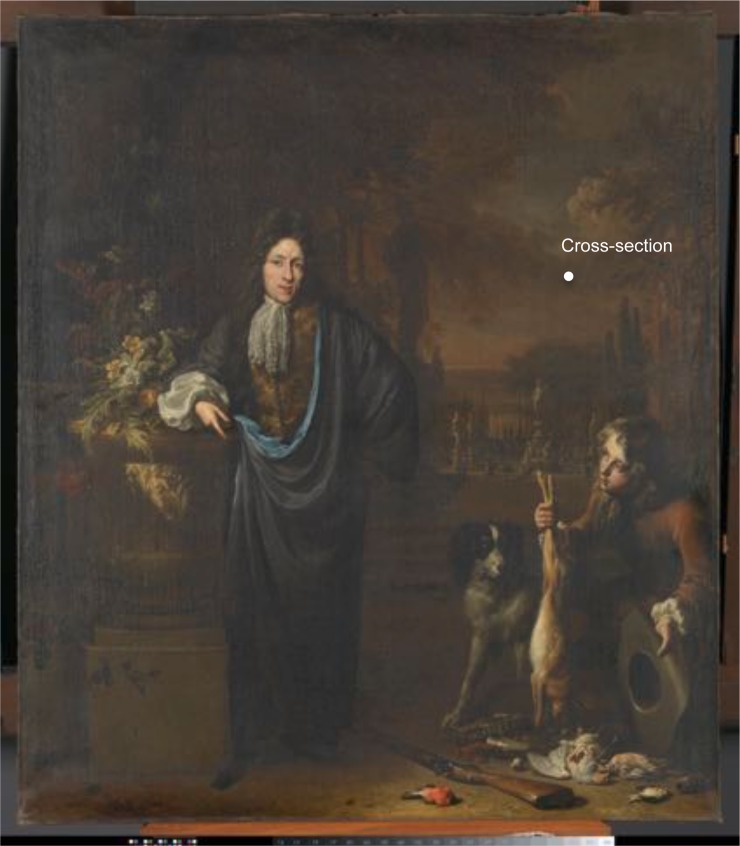
17^*th*^-century Rijksmuseum painting by Jan Baptiste Weenix, the *Portrait of Silvester van Tongeren* (1680-1719, oil on canvas,126.5 cm x 109.5 cm, SK-A-4958), and location of the sample considered for the nanoindentation protocol (Courtesy Rijksmuseum).

**Figure 3:**
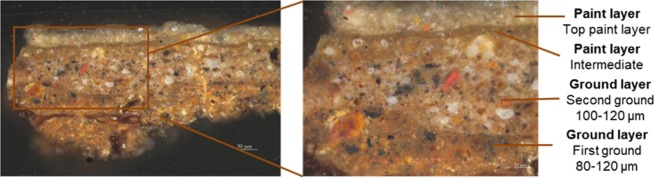
Cross-section taken from Weenix’ *Portait of Silvester van Tongeren*, where four layers are identified (Courtesy Rijksmuseum).

To demonstrate the mapping capability of the instrument, we studied a sample from a 17^*th*^-century painting by Jan Baptiste Weenix, the *Portrait of Silvester van Tongeren*, (oil on canvas,126.5 cm x 109.5 cm, SK-A-4958), from the Rijksmuseum collection (Fig. 2). The paint sample was embedded in a Technovit^©^ 2000 LC methacrylate resin and cured in a Technotray CU blue light polymerization device. The paint sample was dry polished carefully with a sample holder and SiC polishing cloths (Micro-Mesh, final step 12000 mesh) to expose a cross-section of the paint stratigraphy and provide a smooth and flat surface (Fig. 3). The selected paint sample measures 400 *μm* by 700 *μm*, with individual layers from 30 *μm* to 200 *μm* thick. The stratigraphy consists of two ground layers, followed by two paint layers (Fig. 3). The first reddish brown ground layer on that sample mostly comprises earth pigments. The second ground layer is an heterogeneous mixture of earth pigments, bone black and lead white in linseed oil. On top of these preparatory layers, a thin binding medium rich layer containing small amounts of earth pigments, was applied. The fourth layer has a similar composition with some added chalk.

## Results

### Measurements of non-linear viscoelastic behaviour of the grey ground layer

**Figure 4:**
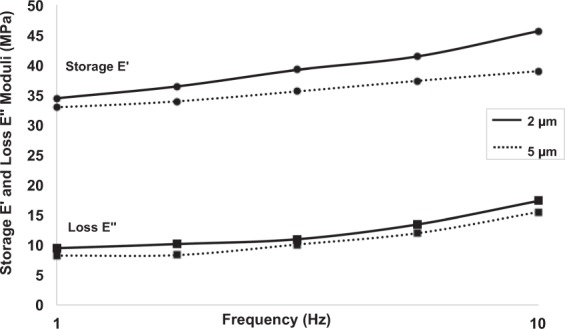
Storage and loss moduli of the lead white grey layer at 2 *μm* and 5 *μm* indentation depths, measured along a frequency sweep profile of 5 measurements from 1 Hz to 10 Hz at an amplitude of A = 0.05 *μm*; the increase of the moduli with the frequency indicates the viscoelastic behaviour and the decrease of the moduli with strain illustrates the non-linearity of the properties.

After adjusting the indentation protocol and the design of the indentation probe to the high stiffness of the laboratory reconstructions (see Methods section), we carried out depth-controlled dynamic indentation measurements (DMA) to evaluate the frequency-dependent storage *E*′ and loss *E*″ moduli of the grey ground, presented in Fig. 4, at two different indentation depths.

At both the indentation depths ${d}_{ia}=2\,\mu m$ and ${d}_{ib}=5\,\mu m$, the frequency sweep induces an increase of the storage modulus, illustrating a stiffening of the material with frequency (Fig. 4). The frequency sweep and resulting moduli confirm the viscoelastic behaviour of the lead white layer, and quantify the elastic and viscous components for this paint film. Then, the non-linearity of the time-dependent behaviour of the lead white layer is highlighted by the slight decrease of both storage and loss moduli at 5 *μm*.

To validate our method, we repeated the measurements indenting the sample from the side as well (Fig. 1). The results obtained on the grey ground containing lead white are comparable with those obtained while indenting the outmost surface, with average storage modulus ${E{\prime} }_{{f}_{1}}$ = 39 MPa and loss modulus ${E{\prime\prime} }_{{f}_{1}}$ = 16 MPa.

**Figure 5:**
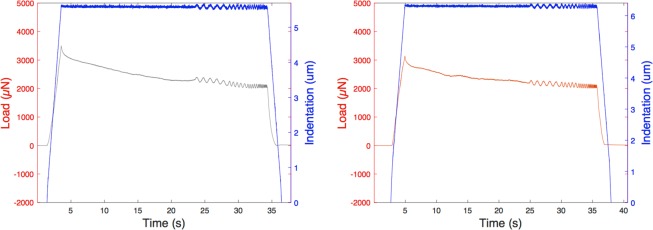
Depth-controlled indentation profile and load for the lead white grey and the red ground layers during DMA analysis on the side of the sample.

Having access to the sample from the side, we further measured the viscoelastic properties of the red ground layer and of the sized canvas. At ${d}_{ia}=2\,\mu m$, we found ${E{\prime} }_{{f}_{1}}$ = 42 MPa and ${E{\prime\prime} }_{{f}_{1}}$ = 12 MPa moduli for the red ground (Fig. 5), and ${E{\prime} }_{{f}_{1}}$ = 70 MPa and loss ${E{\prime\prime} }_{{f}_{1}}$ = 12 MPa for the canvas. Results for the frequency sweep of the red ground are presented in Fig. 5. A non-linear viscoelastic behaviour is also observed for that layer.

### Map of the painting layers modulus

**Figure 6:**
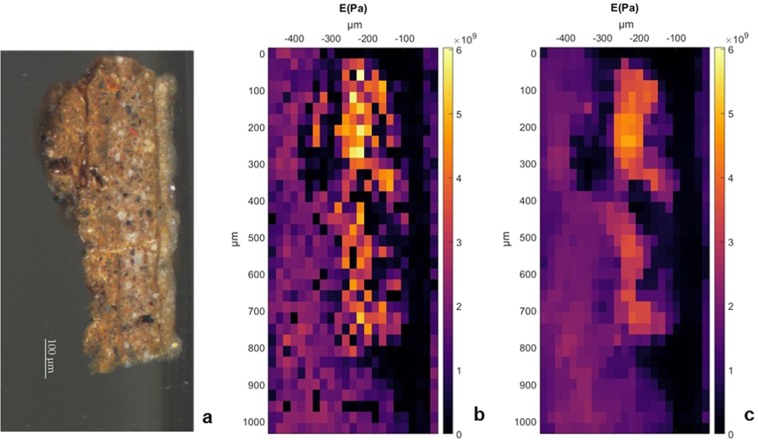
Map of the elastic storage modulus of the sample from the *Portrait of Silvester van Tongeren* by Jan Baptiste Weenix (Rijksmuseum) embedded in resin: (**a**) microscope observation of the cross-section (Courtesy Rijksmuseum) - this integral surface was indented using a 20 *μ*m over 30 *μ*m pitch grid -, (**b**) mapping gathering all the results of the depth-controlled nanoindentation measurements, (**c**) average mapping for three measurements.

To map the mechanical properties of the ${17}^{th}$-century painting, we opted for linear indentation stroke and Hertz model analysis^7,24^ (see Methods section). This method is relatively fast and provides the instantaneous elastic modulus. Figure 6 shows the map obtained following this method on a $20\,\mu m$ by $30\,\mu m$ pitch grid. Clearly, the map nicely overlaps with the morphological features observed on the microscope image, indicating that the ground and paint layers do have, as expected, different mechanical properties. The mismatch of elastic properties between the layers, highlighted by the mapping and the smooth averaging, can then be considered to understand the stresses induced on the layers. The cracking and delamination mechanisms in that painting are part of another research.

It is further evident that the elastic moduli measured on this sample are much larger than those measured on the mock-ups. This result, which has been already observed by others^4,23^, seems to confirm the substantial impact of natural aging on the elastic behaviour of historical paintings. Pigments-oils systems’ evolution over centuries implies a stiffening of all the layers.

**Figure 7:**
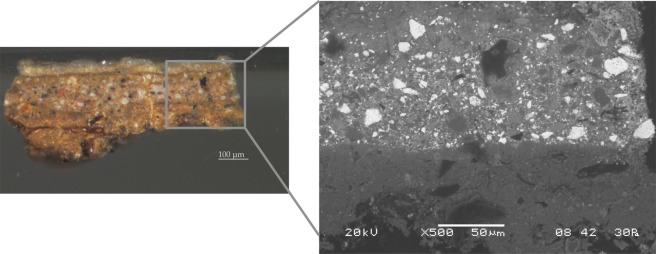
Observation of the cross-section from the *Portrait of Silvester van Tongeren* by Jan Baptiste Weenix (Rijksmuseum) surface that has been indented using a 20 *μ*m over 30 *μ*m pitch grid and SEM backscattered image of a detail of this surface after the static low-depth nanoindentation protocol: no imprint on the surface has modified the sample (Courtesy Rijksmuseum).

Finally, it is important to stress that our indentation protocol does not induce any damage to the sample. Figure 7, which reports SEM images of the sample after indentation, demonstrates that the material has been preserved intact and can be used for other investigations.

## Discussion

The nanoindentation protocol developed in this study allows the evaluation of the mechanical behaviour of laboratory paint reconstructions and historical oil paintings. The protocol defined for each type of sample guarantees a constant indentation depth and indentation speed along the measurements, and allows dynamic analysis of the mechanical properties of the laboratory reconstructions. This methodology provides better stability during the analyses and thus more accurate results than those obtained with piezo-control atomic force microscopy experiments. The indentation depths proposed in the protocols maintain the deformation fields within the boundaries necessary to apply for the theories considered^25–27^.

Depth-controlled indentation profile quantifies the properties of the paint reconstruction stratigraphy. The laboratory reconstructions, comprising a lead white containing layer and a layer with earth pigments applied on a sized canvas, mimick a traditional ${17}^{th}$-century paint stratigraphy. The increase of both elastic and viscous components with the frequency increase illustrates the viscoelastic behaviour of these composite materials. A decrease of the moduli with strain of the lead white grey and red ground layers, indicating a non-linear behaviour, is also observed.

The adapted methodology supports the study of the overall structure of the paint or the reconstruction. Thanks to the investigation on the surface layer, the underneath layers and the sized canvas, this protocol enables a broader evaluation of the behaviour of each individual layer, especially under environmental variations, and provides data on the mismatch in the elastic behaviour, which can lead to cracks and delamination of the painting.

It should also be noted that the results are obtained under a specific protocol, that combines specific approach speed, indentation depth and frequency sweep. Because of the highly non-linear behaviour of the material, a comparison of the values measured with other settings has to be handled carefully. It is important to recognize that this is not a limitation of the instrument, but is due to the intrinsic nature of the sample. Actually, the measurements reported above demonstrate that the instrument is capable of detecting non-linear behaviours that are often neglected in the literature.

To demonstrate the feasibility of the measurement on historical paintings, we adapted a specific depth-controlled indentation protocol to map the cross-section of a sample from the *Portrait of Silvester van Tongeren* by Jan Baptiste Weenix (Rijksmuseum). The elasticity mapping at 0.15 $\mu m$ indentation depth improves the knowledge and the understanding required to a more accurate evaluation of the painting’s behaviour. This research reveals the mismatch between the elastic components of the four identified layers (Fig. 6) as well as the local heterogeneity, thanks to the identification of the difference between the particles and the matrix of the paint film. Moreover, the protocol used preserves the integrity of the material’s surface, enabling a proper evaluation of the behaviour at the interface between the layers.

We believe that this study represents a significant step forward in the field of cultural heritage research, as it paves the way for more accurate, non-destructive laboratory analyses of the viscoelastic properties of historical paintings. In the longer term, it may be even possible to mount the instrument on a robotic arm to allow in situ investigations.

## Methods

**Figure 8:**
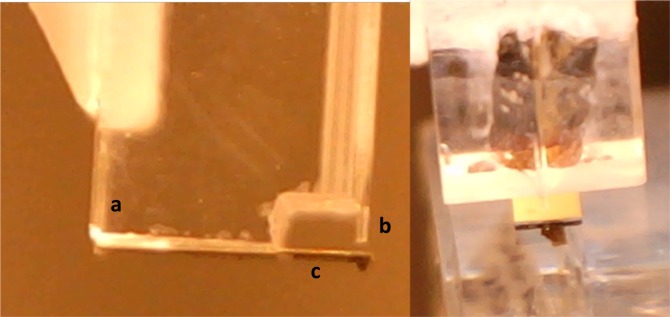
Ferrule-top indentation probe where a glass sphere is glued on top of a cantilever (**c**) attached on a glass ferrule (**a**), using an optical fiber as a readout instrument (**b**), and illustration of the probe on top of the cross-section from the *Portrait of Silvester van Tongeren* by Jan Baptiste Weenix (Rijksmuseum).

Nanoindentation is a local contact method used to quantify the mechanical behaviour of a material. Using a tip with a known geometry, one can apply a deformation while controlling the load or the penetration depth within the sample. In the instrument presented here, the load is brought via a borosilicate sphere glued to the hanging end of a cantilever spring, whose deflection is monitored by means of an optical fiber Fabry-Pérot interferometer, as already amply documented in the literature^10–12,15,28^ (see Fig. 8). When the sphere is in contact with the sample, the cantilever bends according to its own stiffness and the sample’s one. Thus, the material stiffness can be determined by measuring the cantilever bending. Indentation profile at controlled depth is possible thanks to a feedback loop, which supports a constant indentation depth during the whole measurement, while the load is continuously monitored (Fig. 5). A camera placed on top of the equipment allows the proper localisation of the measurement on the material.

To adjust the indentation protocol to the much higher stiffness of the indented samples, we modified the geometry of the cantilever spring to obtain a much higher spring constant ${K}_{cant}$ with respect to the spring constant of the cantilevers used for mechanobiology experiments. Using thicker and shorter mechanical beams, we were able to fabricate two springs with ${K}_{cant}$ of respectively 890 $N\,.\,{m}^{-1}$ and 1100 $N\,.\,{m}^{-1}$, which were then used in the experiments reported above.

The stiffness of each probe was measured applying the calibration methodology described in^29^. The sphere used as non-destructive tip on these cultural heritage objects were respectively 35 $\mu$m and 32 $\mu$m in radius. These relatively small radii allow one to perform the analyses at local scale and to discriminate the differences between the binding medium and the particles observed on some layers (Figs. 3 and 8).

To characterize the material’s behaviour, we could resort to either a static indentation protocol or to a dynamic analysis. For the latter, after a loading at controlled depth and speed, and a relaxation period, small oscillations are applied along a frequency sweep. If present, the phase lag between the load and the deformation illustrates the amount of energy dissipated. Such dynamic analysis highlights the viscoelastic behaviour and provides the storage ${E}^{\text{'}}$ and loss ${E}^{{\prime\prime} }$ moduli characterizing the material.

Indentation measurements were performed in a room under controlled conditions at fixed temperature T = 20 °C and fixed relative humidity RH = 50%. To investigate the mechanical behaviour of paintings cross-sections and reconstructions through the most adequate protocol, different indentation profiles were tested.

### Dynamic analysis on reconstruction paints

The evaluation of the viscoelastic properties of the mock-ups was performed via a depth-controlled dynamic analysis. After touching the surface at an approach speed of $v=5\,\mu m\mathrm{}.{s}^{-1}$, a first loading of 2 seconds reaches the indentation depth ${d}_{i}$ selected. A relaxation time of 20 seconds lets the material recover in order to measure the properties in an equilibrium state. Small oscillations are then performed around the indentation depth. An unloading during 2 seconds finishes the analysis.

The oscillations at an A = 0.05 $\mu m$ amplitude are applied around the indentation depth during a frequency sweep of five frequencies ranging from ${f}_{1}$ = 1 Hz to ${f}_{5}$ = 10 Hz. In this research, two indentation depths have been selected: ${d}_{ia}=2\,\mu m$ and ${d}_{ib}=5\,\mu m$. They both satisfy the small strain approximation required to fit the model validity. At these two controlled depths, dynamic analysis allows the evaluation of the storage ${E}^{\text{'}}$ and loss ${E}^{{\prime\prime} }$ moduli of the materials, the characterization of the elastic or viscoelastic behaviour, as well as the potential non-linearity.

These dynamic analyses were first performed on the outmost surface of the reconstruction, the grey layer (Fig. 1), and repeated analyses on the side of the piece, on the same grey ground layer, to validate the protocol on both directions of the sample. We then investigated the properties of the second layer denominated as red ground layer, and of the sized canvas from the side, to characterize the complete build-up. For each layer and orientation, ten measurements randomly chosen on the reconstruction were performed to verify reproducibility at both ${d}_{ia}=2\,\mu m$ and ${d}_{ib}=5\,\mu m$.

### Static analysis on cross-sections

As expected because of the high stiffness of the 17^*th*^-century paintings^23^, the oscillations required for dynamic analysis on the cross-section surface were not achievable. We adapted our protocol to harvest a depth-controlled static indentation profile, and evaluate the properties through the Hertz model^24^.

In this protocol, a 2 seconds loading up to ${d}_{ic}=0.15\,\mu m$ after the detection of the surface is followed by a 2 seconds unloading. To map the complete cross-section and to differentiate the mechanical behaviour of the four layers previously described, an automatic mapping performs 25 measurements every $20\,\mu m$ along the X axis and 35 measurements every $30\,\mu m$ along the Y axis. The analyses gather a complete $500\,\mu m$ by $1050\,\mu m$ mapping, including the painting sample and the embedding resin.

## Data Availability

The data analysed during the current study are available from the corresponding author on reasonable request.
